# In vivo tracking on longer retention of transplanted myocardin gene-modified adipose-derived stem cells to improve erectile dysfunction in diabetic rats

**DOI:** 10.1186/s13287-019-1325-7

**Published:** 2019-07-16

**Authors:** Hai-Bo Zhang, Feng-Zhi Chen, Shu-Hua He, Yan-Bing Liang, Zhi-Qiang Wang, Li Wang, Ze-Rong Chen, Wei Ding, Shan-Chao Zhao, An-Yang Wei

**Affiliations:** 1 Department of Urology, Nanfang Hospital, Southern Medical University, North of Guangzhou Avenue 1838#, Guangzhou, China; 2 Department of Urology, Shenzhen People’s Hospital, The Second Clinical Medical College of Jinan University, The First Affiliated Hospital of Southern University of Science and Technology, Shenzhen, China; 3 Department of Urology, The Fifth Affiliated Hospital of Guangzhou Medical University, Guangzhou, China; 4 Department of Urology, Tungwah Hospital, Dongguan, China; 5 Department of Urology, The First Affiliated Hospital of Guiyang University of Chinese Medicine, Guiyang, China

**Keywords:** Erectile dysfunction (ED), Adipose-derived stem cells (ASCs), Myocardin, Cell tracking, Diabetes mellitus

## Abstract

**Background:**

Stem cell therapy has revealed a promising future for treating erectile dysfunction (ED), but the fate and curative mechanism of intracavernosal transplanted stem cells are under further exploration. This study aimed to demonstrate the effects of myocardin gene modification on improving erectile function and prolonging the retention of implanted adipose-derived stem cells (ASCs) using in vivo small animal imaging.

**Methods:**

ASCs were isolated, cultured, and identified by flow cytometry and osteogenic and adipogenic induction. The effects of gene modification on cell proliferation, apoptosis, and contraction were determined by CCK-8, EdU, flow cytometry, and collagen gel lattice contraction assays as well as confocal microscopy. A total of 20 normal and 60 diabetes mellitus ED to (DMED) Sprague–Dawley rats were recruited to the 7 day and 21 day groups. Each group contained subgroups of 10 rats each: the negative control (NC), DMED + ASCs plus Ad-Luc-Myocardin, DMED + ASCs plus Ad-Luc, and DMED + phosphate buffer solution (PBS) groups. Erectile function was evaluated with the intracavernosal pressure/mean arterial pressure (△ICP/MAP) ratio. In vivo small animal imaging and an EdU cell tracking strategy were introduced to detect the transplanted ASCs, and IHC and WB were performed to assess smooth muscle cell protein levels.

**Results:**

The ASCs expressed high CD29 and CD90 and scant CD45, while the multi-induction potential was verified by oil red O and alizarin red staining. Gene transfection of myocardin had no significant influence on ASC apoptosis but inhibited cell proliferation and promoted cell contraction. Myocardin combined with ASCs enhanced the therapeutic potential of ASCs for improving the △ICP/MAP ratio as well as α-SMA and calponin expression. In vivo imaging confirmed that ASCs resided within the cavernous body in 21 days, while only a few red EdU dots were detected.

**Conclusions:**

Myocardin induced ASC differentiation towards smooth muscle-like cells and enhanced the therapeutic potential of ASCs for ameliorating ED in STZ-induced diabetic rats. Notably, in vivo small animal tracking was an effective strategy for monitoring the implanted stem cells, and this strategy might have advantages over traditional EdU assays.

**Electronic supplementary material:**

The online version of this article (10.1186/s13287-019-1325-7) contains supplementary material, which is available to authorized users.

## Background

Erectile dysfunction (ED) affects an estimated 35–90% of men with diabetes mellitus (DM) [1], and current treatment strategies, such as phosphodiesterase 5 inhibitors (PDE5i) and vacuum constriction devices, reveal insufficient effects or limitations [2]. In recent years, animal experiments and clinical trials have shown promising therapeutic effects of stem cell transplantation on ED [3]. However, the literature has indicated a large difference in the retaining period of intracavernously injected cells using 5-ethynyl-2′-deoxyuridine (EdU) or other cell trackers in tissue sections [4], and this period differed from less than 5 days to more than 4 weeks [5, 6]. Thus, several issues should be addressed to clarify the cell fate of transplanted stem cells [7]. First, could the injected cells reside within the cavernous body? Second, should injections be repeated to replenish the cells? Finally, is there be any difference between different methodologies? Recently, it was reported that repeat treatments did not provide any benefit for the recovery of erectile function and histomorphometric changes [8], and a single injection showed long-term improvements in ED [9]. Here, we introduce an in vivo strategy, in vivo small animal imaging, which is often applied for the detection of metastatic tumors [10], to track transplanted ASCs; we also compared this technique with the traditional EdU method.

Corpus cavernosum smooth muscle (CCSM) cells provide structural support, and their well-functioning dilation can control blood flow into the corpora, playing a vital role during penile erection [11]. Simultaneously, alleviation of CCSM apoptosis ameliorates ED in rats [12], suggesting the efficiency of this curative strategy for improving CCSM cell function. Among the regulators, myocardin is known to be required for the maintenance of functions in smooth muscle cells [13, 14], and it was demonstrated to enhance the therapeutic potential for myocardial infarction in mesenchymal stem cells in an animal model [15]. We have previously demonstrated the efficiency of myocardin gene therapy in ED in a rat model of bilateral cavernous nerve injury [16], but the effects of the gene combination with adipose-derived stem cells (ASCs) remain to be clarified. This study aims to prove the myocardin genetic modification of ASCs to improve erectile dysfunction in diabetic rats.

## Methods

### Experimental design

All experimental male Sprague–Dawley (SD) rats weighed 250–300 g were purchased and housed in the Experimental Animal Center of Nanfang Hospital, Southern Medical University of China. All experimental protocols were performed under the Institutional Animal Care and Use Committee-approved guidelines at our institution.

The animals were designated into the 7 day and 21 day groups. Each group was divided into 4 subgroups: the negative control (NC), diabetic mellitus ED (DMED) + ASCs plus Ad-Luc-Myocardin, DMED + ASCs plus Ad-Luc, and DMED + PBS groups (*n* = 10 per group). The DMED rats were induced with streptozotocin and screened by the apomorphine-induced erection test to confirm ED before subsequent one-time intracavernosal injection of 50 μL PBS with 1 × 10^6^ cells or PBS only. After 7 or 21 days, all rats were anesthetized for in vivo small animal imaging before intracavernosal pressure (ICP) and mean arterial pressure (MAP) tests. The penises of the rats in the 21 day group were harvested, weighed, and processed for further study.

### Apomorphine-induced erection test

An apomorphine-induced erection test was performed as we previously described [17]. Rats were first moved to a tranquil and dimly lit laboratory and set in a separate transparent observation kit at least 10 min before any operation to allow them to adapt to the surroundings. Then, a single subcutaneous injection of 100 μg/kg apomorphine (APO, Sigma, USA) was given via the loose skin at the back of the neck. During the following 30 min, the status and frequency of penile erection in rats were observed by two trained technicians, and each instance of glans engorgement or the appearance of the penile shaft represented one erection. Finally, rats with no erection were defined as having ED and were included in the subsequent experiments.

### In vivo small animal imaging

In vivo tracking of the transfected ASCs was performed using the IVIS Lumina II system as previously reported [10]. Briefly, all groups of animals were intraperitoneally injected with 150 mg/kg D-luciferin (Bioworld, Minneapolis, MN, USA) dissolved in DPBS (HyClone, GE, Boston, USA) at a concentration of 15 mg/ml for 5 min before anesthesia. Then, animals were placed in the camera apparatus, and local images were taken.

### Erectile function evaluation

Erectile function was assessed with the △ICP/MAP ratio as previously described [16]. Rats were sterilized and anesthetized, and a low abdominal incision was made. The cavernous nerves were exposed for stimulation with a bipolar, stainless steel electrode. Subsequently, a 25-G needle containing 100 U/ml heparin solution was inserted into the right penile crus, which was connected to the transducer and amplifier of the MP150 biopac system (Biopac Systems Inc., CA, USA) and supporting software AcqKnowledge® V4.4. The stimulus parameters were as follows: amplitude (5 mA), frequency (20 Hz), pulse width (0.2 ms), and duration (60 s). The erectile evaluation consisted of measuring basal ICP, maximal ICP, change in ICP (ΔICP), and ΔICP/MAP.

### EdU labeling and detection

EdU (RiboBio Co., Guangzhou, China) was introduced for cell labeling and tracking [18]. Passage 2 ASCs were incubated with 10 mM EdU for 24 h before the final injections. Afterwards, the transplanted and labeled ASCs were detected by tissue immunofluorescence with red fluorescence Apollo® 567, along with Hoechst 33342 for indication of the nucleus.

### Cell culture and processing

Primary ASCs were obtained as previously described [19]. Fat tissues in the inguinal area from SD rats weighing 180–220 g were isolated, minced in digestive solution containing 0.15% type I collagenase, and incubated in a 120 r/min 37 °C constant temperature hybridization oven (UVP, Upland, CA, USA) for 1 h. Subsequently, the jelly-like-digested adipose tissues were resuspended with 10% FBS (HyClone, GE, Boston, USA) complete medium to terminate the digestion and were washed with PBS in a 1200 r/min centrifuge, followed by processing with FACS Lysing Solution for 10 min. The final precipitate was then collected, and passage 3 cells were used for adipogenic and osteogenic inductions using corresponding inducing medium (PanEra laboratories. Inc., Beijing, China) for 21 days to confirm the multipotential differentiation capability.

### Flow cytometry assay

Apoptotic cells were evaluated by annexin-V fluorescein isothiocyanate and a propidium iodide Apoptosis Detection Kit (Dojindo, Japan) according to the manufacturer’s protocol. Stained cells were then analyzed with a FACScan flow cytometer (BD, NJ, USA).

### Adenovirus production and infection

Adenovirus particles carrying the pHBAd-MCMV-RLuc vector with the myocardin precursor or an empty vector were constructed by Hanbio Co. LTD (Shanghai, China). ASCs were infected with adenovirus at a multiplicity of infection of 50.

### CCK8 and EdU cell proliferative assay

A Cell Counting Kit (CCK8, Dojindo, Japan) and 5-ethynal-2′-deoxyuridine (EdU, RiboBio, China) assays were performed according to the manufacturer’s instruction to verify the proliferative capacities. For the CCK8 test, cell proliferative rates were determined by OD values read with a multimode microplate reader 0, 2, 4, 6, 12, 24, and 48 h after gene or empty vector stimulation. On the other hand, in the EdU reaction, proliferating cells were double stained with Hoechst 33342 (blue) and EdU (red), while quiescent cells were double stained with Hoechst only. The total and EdU+ cells were counted in 3 independent fields under a × 400 microscope in every cultured well. Time growth curves were fabricated with GraphPad Prism 5 software.

### Collagen gel lattice contraction assay

A modified in vitro cell contractility assay was carried out as we demonstrated before [16]. Briefly, ASCs with different treatments at a density of 2 × 10^5^ cells/ml were mixed with solubilized type I collagen (Sigma, USA) to form a cell–collagen suspension at a final concentration of 1 mg/ml. A total of 200 μl of suspension was dropped in a 35-mm culture dish immediately and then incubated in a growth medium for another 5 days before the cell–collagen lattice was mechanically released from the underlying plastic substratum. Subsequently, the lattice was exposed to serum-free Dulbecco’s modified Eagle’s medium (DMEM), DMEM plus 10% fetal bovine serum (FBS), or DMEM plus 1 μM calcium ionophore (Ca-Ionophore, Sigma, USA). Serum-free DMEM was used as a negative control, while FBS was used as a positive control. The diameters before releasing the lattice and 10 min after the various exposures were recorded for relative percent contraction.

### qRT-PCR

Total RNA was extracted from ASCs using RNAiso plus reagent (Takara, Japan) according to the manufacturer’s instructions. qRT-PCR was performed using the LightCycler® 480 II (Roche, Basel, Switzerland) with the SYBR Green PCR kit (Takara, Japan). Gene β-actin was used as an internal control. The specificity of the amplification products was confirmed by melting curve analysis. Triplicate samples were analyzed in three independent experiments. Primer sequences are shown in Additional file 1: Table S1.

### Western blotting

Protein samples were obtained from ASCs on ice and from penile tissues in liquid nitrogen with radioimmunoprecipitation assay buffer (Cell Signaling Technology, CST, MA, USA) containing protease and phosphatase inhibitors (Roche, Switzerland). The protein concentration was determined by the Bradford method using the BCA Protein Assay (Thermo Fisher Scientific Inc., MA, USA). Protein lysates were separated on an 8–10% sodium dodecyl sulfate-polyacrylamide gel and electrotransferred to a polyvinylidene difluoride membrane (Bio-Rad, CA, USA) that was blocked with 5% bovine serum albumin for 1 h at room temperature and probed overnight at 4 °C with primary antibodies, followed by incubation with horseradish peroxidase-conjugated secondary antibodies (Abcam, Cambridge, UK). Immunodetection was performed using enhanced chemiluminescence reagent (Bio-Rad, USA). Antibodies against myocardin, 1:400; collagen I, 1:1000 (Abcam, UK); SRF, cleaved-caspase3, caspase3 (all 1:1000, CST, USA); α-SMA, calponin (all 1:400, Santa Cruz, USA); β-tubulin (1:8000, ABclonal, Boston, MA, USA); Bcl-2, Bax, SOX2, OCT4, PCNA (all 1:700, Proteintech, Chicago, IL, USA); and β-actin (1:8000, Ray Antibody Biotech, Beijing, China) were used.

### Immunofluorescent staining and confocal microscopy

Different groups of ASC cells were seeded and grown on glass coverslips before fixation with 4% paraformaldehyde and were incubated with 0.25% Triton X-100/1% bovine serum albumin (BSA)/PBS. The primary antibodies, α-SMA (1:50, Santa Cruz, USA), calponin (1:100, Santa Cruz, USA), myocardin (1:100, Abcam, UK), and SRF (1:50, CST, USA), were incubated at 4 °C overnight, followed by double staining of secondary antibodies (all 1:50, Bioworld, USA) with FITC (green) or TRITC (red) at room temperature and protected from light for 1 h. 4′,6-Diamidino-2-phenylindole (DAPI, Abcam, UK) was introduced to locate the cell nucleus. The contractile proteins α-SMA and calponin and the molecular colocalization of myocardin and SRF were detected by an Olympus laser scanning confocal microscope (Olympus, Shinjuku Monolith, Japan).

### Histology

Freshly dissected tissues were fixed and prepared for histological examinations. H&E and Masson’s trichrome staining as well as immunochemistry (IHC) were performed according to the manufacturer’s instructions. Sections were cut at 4 μm and incubated with myocardin (1:100, Abcam, UK) and α-SMA (1:50, Santa Cruz, USA). Digital images were acquired with an Olympus microscope (Olympus, Shinjuku Monolith, Japan), and the SM-to-collagen ratio of Masson’s trichrome staining was evaluated using Image-Pro Plus 6.0.

### Statistics

Statistical analysis was performed with SPSS software version 21.0 for Windows. The results were recorded as the mean ± SEM. Groups were compared using Student’s *T* test or one-way ANOVA. The univariate general linear model with fixed factors of time and group was performed to test the CCK8 results. The statistical significance was determined at the 5% confidence level (*p* < 0.05).

## Results

### Adipogenic and osteogenic induction and immune phenotype of ASCs

**Fig. 1:**
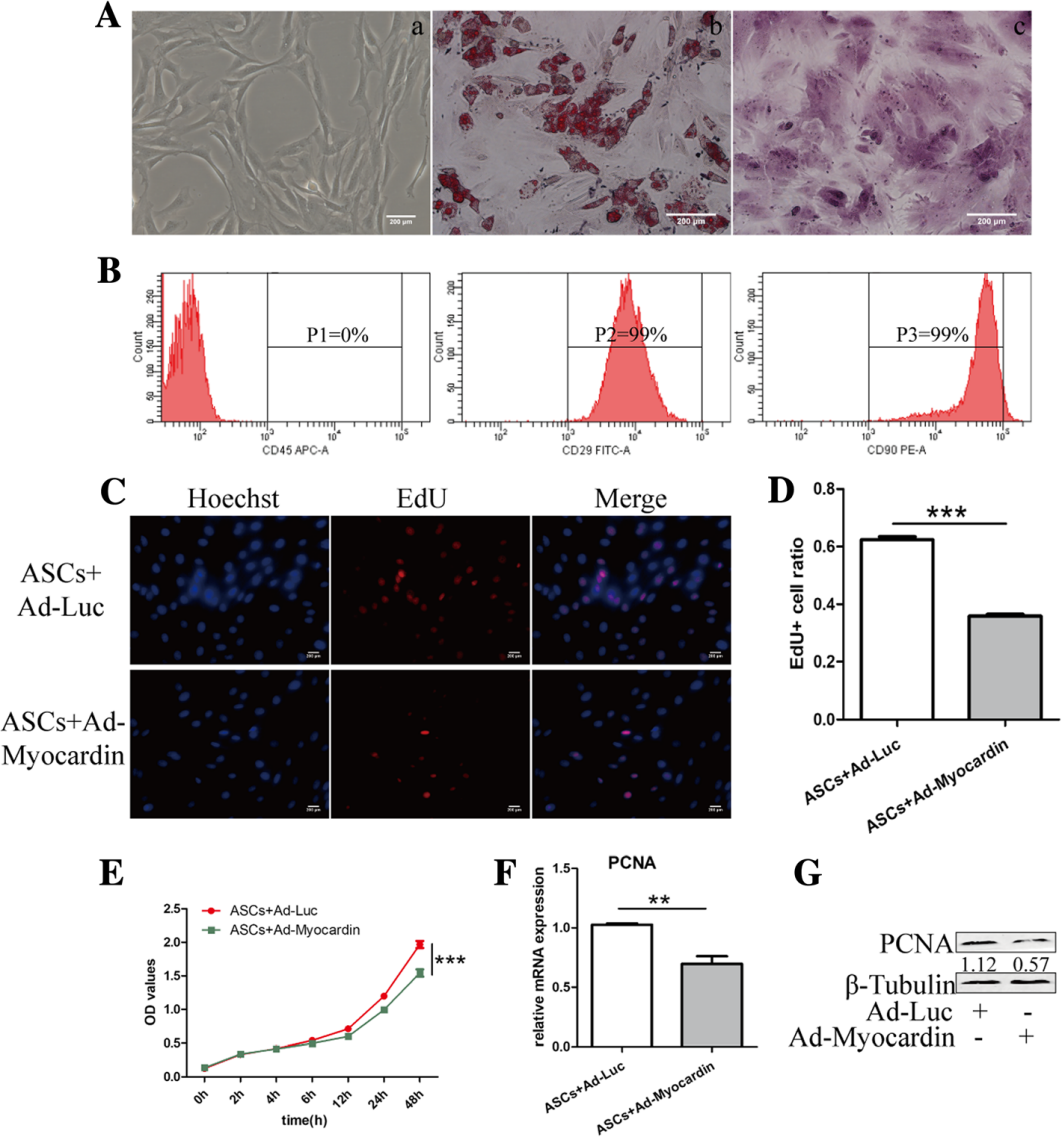
Myocardin reduced the proliferative capacity of ASCs in vitro. **A** Typical cell image (left), adipogenesis and osteogenesis of ASCs confirmed by oil red O (middle) and alizarin red (right) staining under × 200 magnification. **B** CD45 was negatively expressed, while CD29 and CD90 were positively expressed in ASCs. **C** EdU assay to identify the proliferating cells 48 h after stimulus, and the cells were stained red under × 400 magnification. **D** The EdU+ (red) cell ratio was counted, and evident declines were identified in the myocardin-treated group. **E** CCK8 confirmed the proliferative capacity trend of the 2 groups. **F** Proliferating cell nuclear antigen (PCNA) mRNA and **G** protein expression levels were detected using qRT-PCR and western blotting. Scale bar = 200 μm. Cell experiments performed *n* = 3. **p < 0.01; ***p < 0.001

Primary ASCs were isolated and cultured. A typical long fusiform shape with a whirlpool-like growth on the passage 3 cell image is shown in Fig. 1A (a), as well as adipogenesis and osteogenesis images confirmed by oil red O (b) and alizarin red staining (c). Flow cytometry was performed with CD 29, CD90, and CD45 to detect the immune phenotype of the cultured ASCs. As shown in Fig. 1B, CD29 and CD90 were expressed in 99% of cells, while CD45 was negatively expressed.

### The proliferative capacity of ASCs was reduced with overexpression of myocardin

EdU showed that the proliferative cell rates were 35.93 ± 1.42% and 62.38 ± 2.53% in the Ad-myocardin and vector cells, respectively (Fig. 1C, D). Similarly, the CCK-8 assay was performed, and overexpression of myocardin resulted in reduced proliferative capacity within 48 h in ASCs transfected with Ad-myocardin compared with that in ASCs transfected with empty vector (Fig. 1E). Further qRT-PCR and immunoblotting analyses revealed downregulated mRNA and protein expression of the cell proliferation marker PCNA by myocardin (Fig. 1F, G).

### Myocardin induced ASC differentiation towards smooth muscle-like cells

**Fig. 2:**
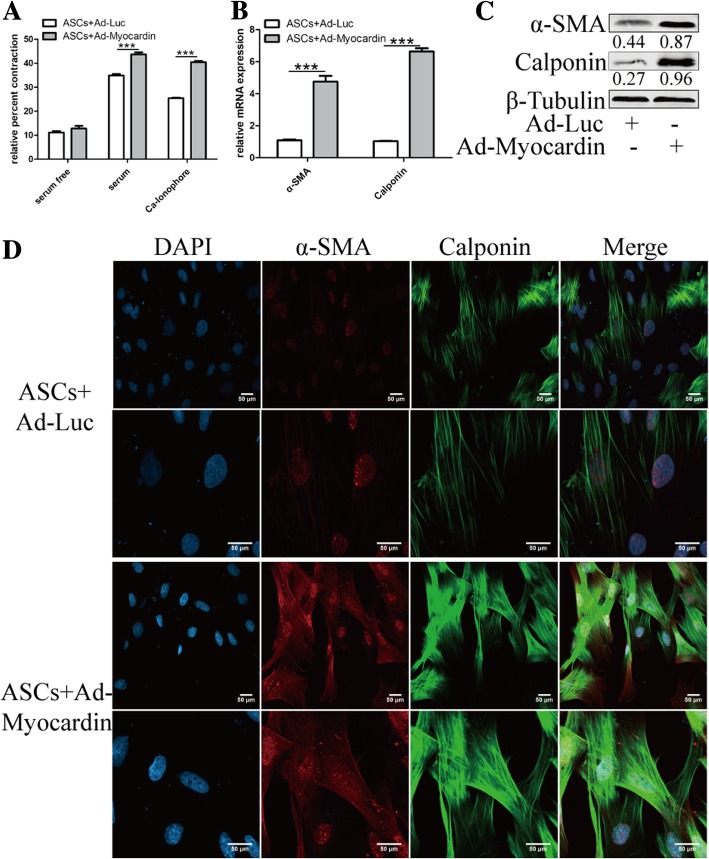
Enhanced contractility of myocardin-transfected ASCs. **a** Cell contraction ratio was increased in both DMEM plus 10% FBS and DMEM plus Ca-ionophore cells treated with myocardin, while there was no difference among groups with DMEM stimulation. **b** The α-SMA and calponin mRNA and **c** protein expression levels were detected using qRT-PCR and western blotting. **d** Overexpression of myocardin presented clearer smooth muscle cell markers α-SMA and calponin. Images obtained with confocal microscopy under × 600 (upper row) and × 1200 (lower row) magnifications. The red rectangle box indicates the zoomed area. Scale bar = 50 μm. Cell experiments performed *n* = 3. ***p < 0.001

The collagen gel lattice contraction assay indicated enhanced cell contractility in the transfected cells, as shown in Fig. 2a. The smooth muscle cell cytoskeleton and contractile proteins α-SMA and calponin were significantly upregulated at the mRNA and protein levels (Fig. 2b, c). Simultaneously, confocal was introduced to detect the intracellular localizations and expression intensities of α-SMA and calponin (Fig. 2d). Taken together, the results show that myocardin induced ASC differentiation towards smooth muscle-like (SML) cells.

### Myocardin interacted with SRF and inhibited SOX2 and OCT4

**Fig. 3:**
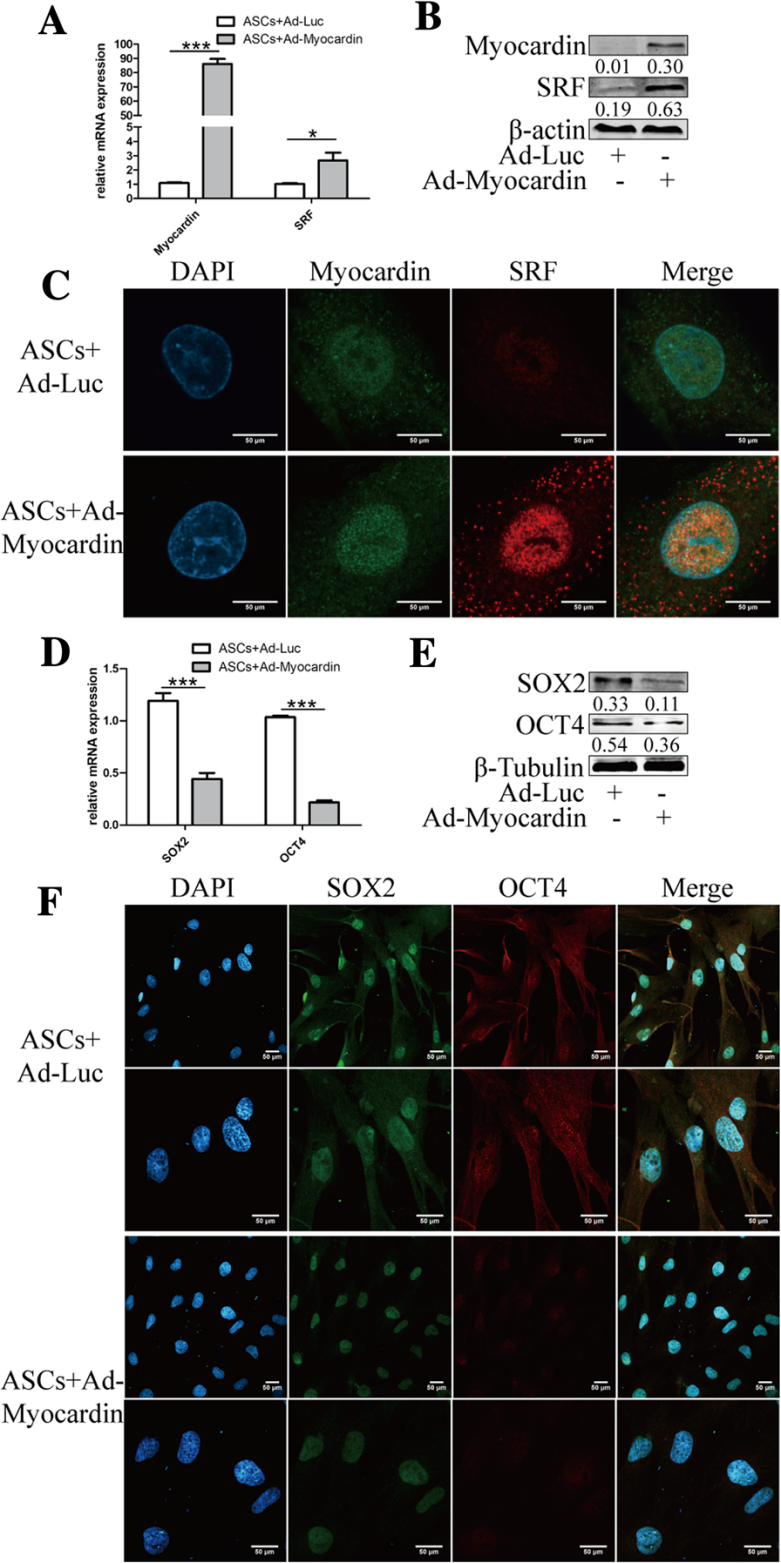
Myocardin promoted SRF and inhibited SOX2 and OCT4. The serum response factor (SRF) was elevated in mRNA (**a**) and protein levels (**b**) along with overexpression of myocardin. **c** The colocalization of Myocardin and SRF was detected in the nucleus under × 2000 magnification with confocal microscopy. The stem cell markers SOX2 and OCT4 were inhibited by myocardin at the mRNA (**d**) and protein levels (**e**), as well as the immunofluorescence intensities indicated by confocal microscopy under × 600 (upper row) and × 1200 (lower row) magnifications (**f**). The red rectangle indicates the zoomed area. Scale bar = 50 μm. Cell experiments performed *n* = 3. **p < 0.01; ***p < 0.001

The classic mechanism of myocardin for the promotion of contractile proteins in smooth muscle cells with SRF was confirmed here in ASCs. qRT-PCT and WB were performed to determine the increasing expression at the mRNA and protein levels by myocardin (Fig. 3a, b). Immunofluorescence confocal imaging was introduced to detect the interaction and colocalization of myocardin and SRF (Fig. 3c). In addition, the general stem cell markers SOX2 and OCT4 were validated to be downregulated in transfected ASCs (Fig. 3d–f), indicating the mechanism by which myocardin induced ASC differentiation towards SML cells by decreasing the ASC markers.

### Gene modification of myocardin prolonged the retention of transplanted ASCs within the cavernous body

**Fig. 4:**
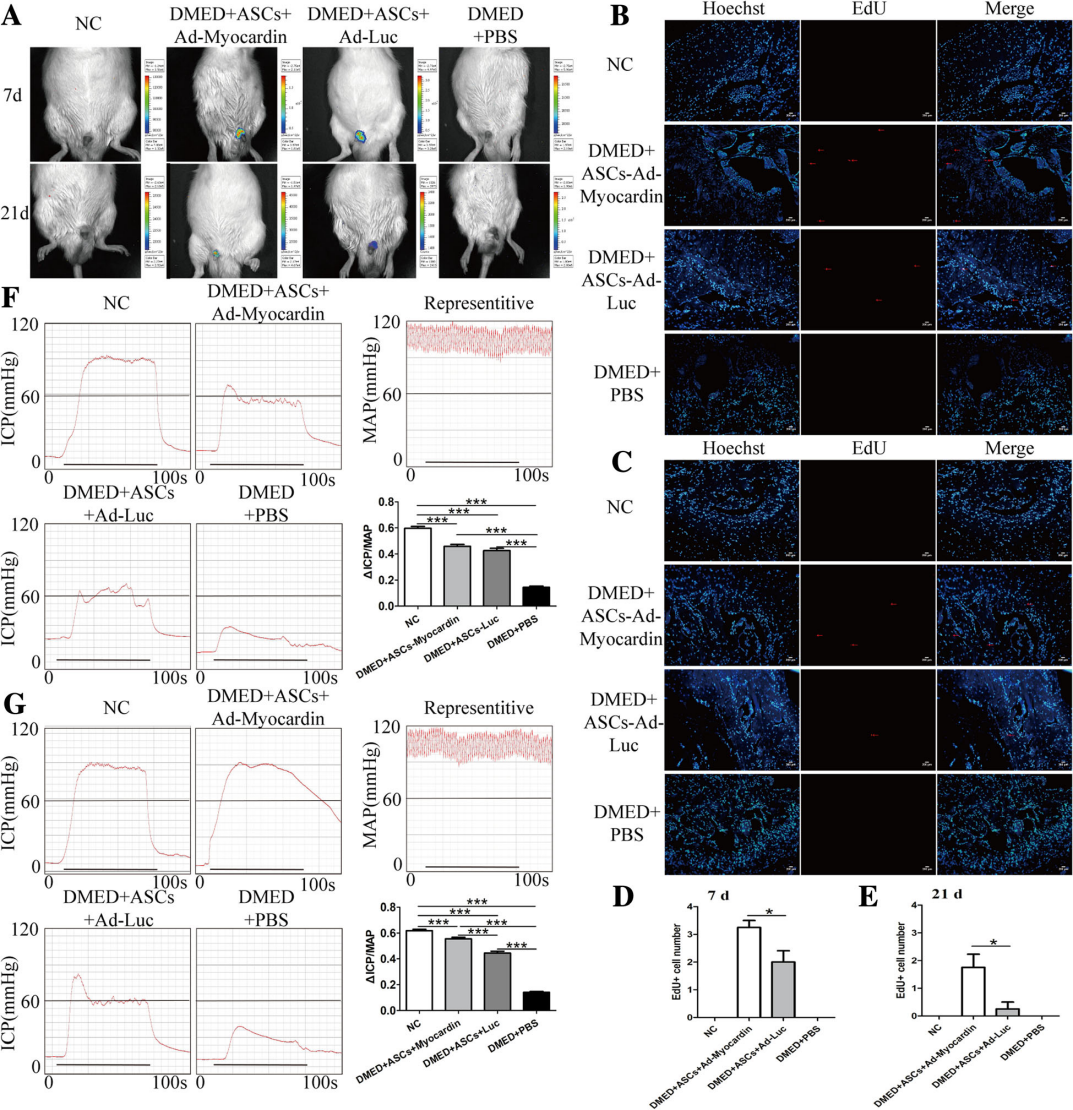
Myocardin prolonged the retention and enhanced the therapeutic potential of transplanted ASCs. **a** In vivo small animal imaging revealed the fluorescence intensities produced by luciferase incorporated into the adenoviruses, indicating the retention amount of ASCs 7 and 21 days post-transplantation. The EdU cell tracking strategy was introduced to detect the implanted ASCs, which were stained red with EdU at 7 (**b**, **d**) and 21 days (**c**, **e**). **f** Representative images of intracavernosal pressure (ICP) and mean arterial pressure (MAP) and the △ICP (maximum ICP – basal ICP)/MAP ratio are shown in the 7 day group as well as the 21 day group of rats (**g**). Animals tested *n* = 10. ***p < 0.001

In vivo small animal imaging and an EdU cell tracking strategy were performed to determine the retention of transplanted ASCs within the cavernous body in different animal groups. Before ASC injection, the EdU transfection efficiency was proven to be 81.53 ± 1.42%, which is in support of subsequent detection (shown in Additional file 2: Figure S1). As shown in Fig. 4a, no fluorescence was detected in NC or DMED+PBS rats of both the 7 day and 21 day groups, which served as controls for luciferase gene transfection. The fluorescence intensities of the two 7 day experimental groups were significantly higher than those of the 21 day groups, revealing a time-dependent decrease in the retention of ASCs, and fluorescence in the DMED+Ad-Luc-Myocardin rats was notably stronger than that in the DMED+Ad-Luc rats in the 7 day and 21 day groups, respectively. Subsequently, frozen sections were made, and EdU+ cells were shown in the different groups (Fig. 4b–e), indicating the longer retention in the gene-modified stem cell groups, which is consistent with the results of in vivo imaging.

### Overexpression of myocardin promoted the therapeutic potential of ASCs in ED

As shown in Fig. 4f and g, the 7 day and 21 day DMED+PBS rats showed a remarkable decline in maximum ICP compared with the NC+PBS rats. The ASC-treated rats showed a significant increase in maximum ICP in all subgroups. There was no difference in the MAP among all the groups. Statistically, the △ICP/MAP values in the two ASC groups were significantly higher than those in the DMED+PBS groups but were still much lower than those in the NC groups. There was no difference between the DMED+Ad-Luc-Myocardin and DMED+Ad-Luc groups at 7 days (Fig. 4f). However, in the 21 day groups, myocardin gene-modified ASCs significantly increased the △ICP/MAP ratio compared with the ASC plus vector group (Fig. 4g).

### CCSM cell function was improved by transplantation

**Fig. 5:**
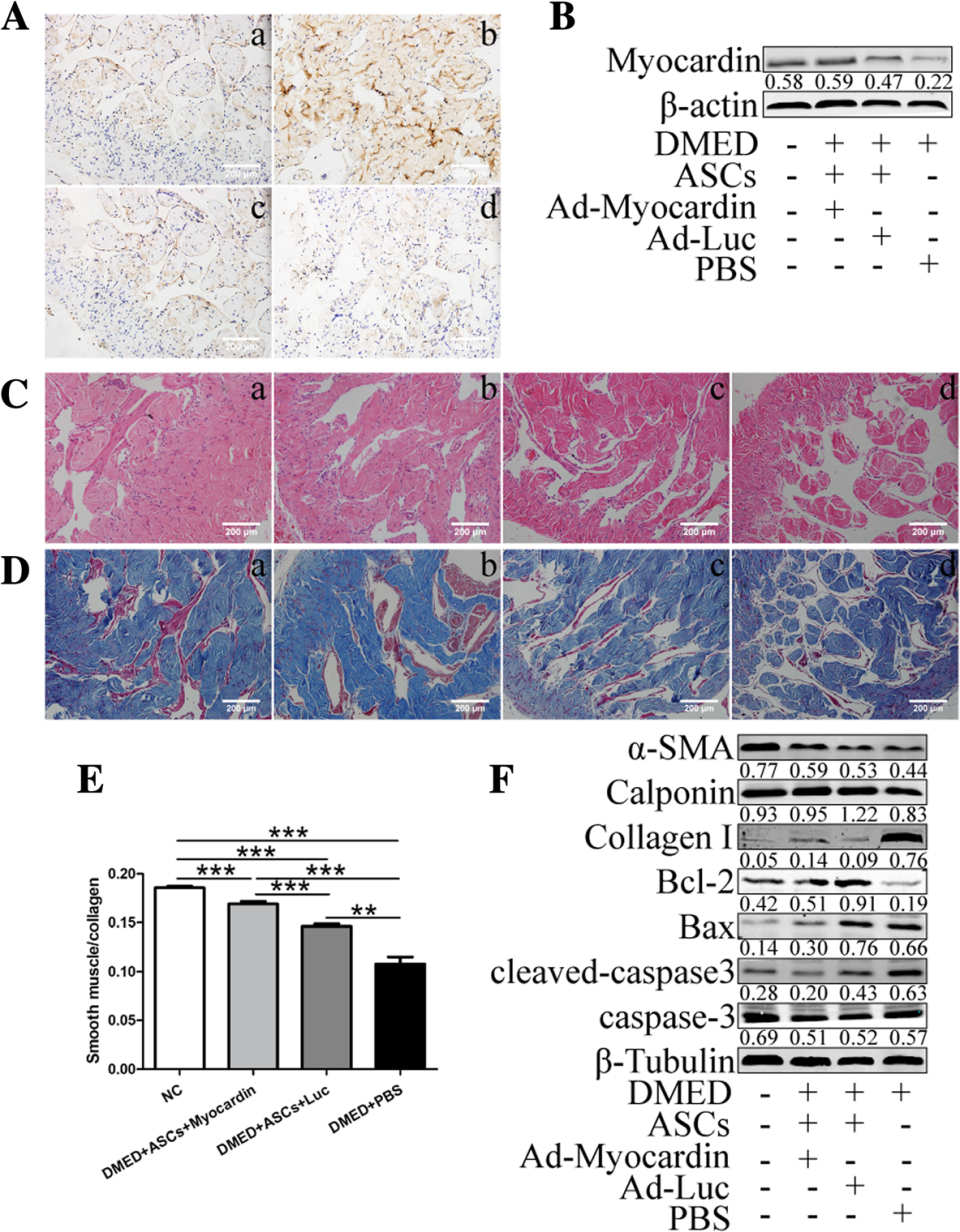
Gene modification improved the effects of ASCs on ameliorating corpus cavernosum smooth muscle (CCSM) functions. IHC (**A**) and western blot (**B**) revealed the higher expression of myocardin in rats with gene-transfected ASCs. **C** A thinner smooth muscle layer and discontinuous and disordered cavernous sinuses were found in all diabetic rats, with more severe changes in the PBS group. **D** In the Masson’s trichrome staining images, the ASC and gene-modified ASC groups retained the SM-to-collagen ratio compared with the PBS group, which is shown in the statistical graph (**E**). **F** The protein expression levels of the smooth muscle markers α-SMA and calponin, fibrosis marker collagen I, anti-apoptosis factor Bcl-2, apoptosis molecule Bax, and cleaved-caspase3 were detected. Scale bar = 200 μm. Animals tested *n* = 10. ***p < 0.001

The transfection efficiency of myocardin was further confirmed using IHC and WB (Fig. 5A, B). The morphological changes and smooth muscle (SM)-to-collagen ratios of the different groups were detected with H&E and Masson’s trichrome staining, as shown in Fig. 5C and D. ASC treatment reduced the damage to morphological changes caused by diabetes mellitus and significantly increased the SM-to-collagen ratio compared with the DMED+PBS group, but the ratio was still lower than that in the NC group (Fig. 5E). In addition, gene modification remarkably increased the therapeutic effects of ASCs. As shown in Fig. 5F, the protein expression levels of myocardin, collagen I, cleaved-caspase 3, and the CCSM cell markers α-SMA and calponin were measured. Taken together, the results show that myocardin overexpression promotes the effect of ASCs on improving the CCSM cell number and functions and inhibits the pathological process of fibrosis and apoptosis.

## Discussion

Over the past few years, efforts have been made to explore novel therapeutic strategies for erectile dysfunction (ED), including stem cell transplantation, gene therapy, and low energy shock wave therapy [20]. Among these strategies, stem cell therapy has demonstrated a substantial curative effect in both animal studies and clinical trials [9, 21]. A meta-analysis by Ji-Hong Liu et al. summarized 10 animal studies containing 302 diabetic ED rats that received a single injection of stem cells and found main effects on increasing the intracavernosal pressure, cavernosal tissue smooth muscle/collagen ratio, and contents of nNOS, eNOS, and VEGF and inhibiting cell apoptosis [22]. However, how stem cells exert the aforementioned effects remains to be clarified. It was speculated that transplanted stem cells were capable of differentiating into corpus cavernosum smooth muscle cells (CCSMCs) or endothelial cells to repair damaged tissues. However, no direct evidence was found to support this transformation [23]. In recent years, researchers have tended to reveal the role of cytokines secreted by mesenchymal stem cells (MSCs) and have demonstrated therapeutic effects by the lysate or conditioned medium of MSCs. Notably, different cytokines were selected in the published studies, and the cytokine profile of the MSCs has not been revealed by medium- or high-throughput screening [24]. Furthermore, not limited to cytokines, other investigators and our team reported previously that exosomes derived from ASCs exerted a measure of benefit compared with ASCs alone [12, 19]. Thus, the mysteries of these miraculous stem cells have yet to be uncovered.

Among the published literature, diverse effects have been observed in different studies, especially on the transplanted stem cell retention period within the cavernous body. Dr. Jong-Ho Won et al. reported that injected bone marrow stem cells reside in the penis and slowly flow into the pelvic area in 3 months using MRI monitoring [25]. Furthermore, Yu-tian Dai et al. found that only a few implanted cells were detectable 4 weeks after injection using EdU cell tracking technology [6]. However, a similar study with similar stem cell tracker NanoShuttle magnetic nanoparticles suggested that no remaining stem cells were detected 9 days post-transplantation [5]. Therefore, it remains to be determined whether injected stem cells remain in the cavernous body or spread rapidly. In this study, we compared two methods for cell tracking using in vivo small animal imaging, which has frequently been applied for tumor measurement and metastatic detection, and tissue immunofluorescence of EdU, demonstrating the superiority of the in vivo strategy over pathological section detection, which required animal execution and cumbersome methods and was known to have the shortcoming of slicing randomness. Our data support evidence of the abundant retention of implanted cells for at least 21 days, which is consistent with other published studies using MRI [4, 25].

Another important issue is the duration of stem cell effects. When applied in clinical trials, this novel cell therapeutic regimen appeared to have diverse potencies with immediate and lasting effects. Yiou Rene et al. suggested a total effective rate of 75% [3], while Haahr et al. reported that 8 of the 17 participants were free of immediate effects 6 months after the regimen [21]; Jong Yoon Bahk et al. demonstrated that 4 of 7 patients returned to the initial state after 9 months [26]. The benefits of stem cell therapy include improvements in the International Index of Erectile Function-15 and Erection Hardness Scale questionnaires as well as peak systolic velocity and penile nitric oxide release test [3, 27]. Notably, almost all patients were in need of other assistive treatments to complete their regimens, such as a phosphodiesterase type 5 inhibitor (PDE5i) [28]. Therefore, to enhance the therapeutic effects of stem cells, two strategies have been implemented that target extending the retention of transplanted cells and improving certain curative capacity by gene modification in animal studies. It has been proven that chemical modification of hydrogel [29] or poly l-glutamic acid (PLGA) membranes [30] increases both the number of labeled stem cells and the intracavernosal pressure, but determining longer effects of these animal experiments and the safety of these synthetic compounds on the human body requires further observation.

In recent years, gene-modified stem cells have attracted unprecedented enthusiasm and were expected to play a stronger role in repairing damaged tissues compared with untreated stem cells [31]. In our study, myocardin, which has been confirmed to be essential for the development and function of smooth muscle cells [32, 33], was introduced to induce the differentiation of ASCs towards SML cells in vitro by interacting with SRF and inhibiting the general stem cell markers SOX2 and OCT4 [34], as well as enhancing therapeutic effects through both prolonging the retention of implanted ASCs and increasing the expression of smooth muscle contents. Notably, gene modification appeared safe and controllable, as it did not increase the cell apoptosis rate or morality in the animal model. It is known that normal erectile function requires coordination among cavernous nerves, vessels, and endothelial and smooth muscle cells, and smooth muscle cells have been shown to be targets of other factors [35, 36]. Thus, in the current research, the focus was on improving smooth muscle function, although it has been demonstrated that stem cells have extensive effects on nerves [37] and endothelial cells [38]. However, the specific mechanism in which myocardin gene modification enhanced the therapeutic potential of ASCs was yet to be uncovered. In our previous study, myocardin was proved to maintain the contractile phenotype of CCSM cells, promote cell contractility, and suppress proliferative capacity in a rat model of bilateral cavernous nerve injury [16]. In the current research, there were two possibilities in the contribution of myocardin to the improvement of erectile dysfunction in diabetic rats. The expression of myocardin was found to be upregulated in the DMED+Ad-Luc-Myocardin group and it was speculated that myocardin exerted similar regulatory effects in DMED rats. On the other hand, higher differentiation efficacy of ASCs induced by myocardin was detected and it might be of benefit for repairing the damaged tissues.

Our study, consistent with other similar experiments or clinical trials, revealed a promising future for stem cells in the treatment of ED. Moreover, we demonstrated the validity of gene-modified stem cells by myocardin and the superiority of an in vivo imaging strategy over traditional EdU tracking. However, limitations included the duration of adenovirus, leading to the longest observation of up to 3 weeks. However, the choice of adenovirus would meet the needs of high transfection efficiency and cotransfection with EdU in the current research. Future efforts could be made to monitor long-term effects using a lentivirus.

## Conclusions

Myocardin induced ASC differentiation towards SML cells and enhanced the therapeutic potential of ASCs for ameliorating ED in diabetic rats. Notably, in vivo small animal tracking was an effective strategy for monitoring implanted stem cells, and this strategy might have advantages over traditional EdU assays.

## Additional files

Additional file 1: **Table S1.** Primer sequences used in this study. The forward and reverse primer sequences of β-actin, PCNA, α-SMA, Calponin, myocardin, SRF, SOX2, and OCT4 are shown in **Table S1**. (DOCX 16 kb)

Additional file 2: **Figure S1.** EdU transfection efficiency was confirmed. Passage 2 ASCs were incubated with 10 mM EdU for 24 h before intracavernous injections and 81.53 ± 1.42% of cells were stained red under × 400 magnification. Scale bar = 200 μm. (TIF 608 kb)

## Data Availability

All data generated or analyzed during this study are included in the published article.

## Abbreviations

ED: Erectile dysfunction
ASCs: Adipose-derived stem cells
CCK-8: Cell Counting Kit
EdU: 5-Ethynyl-2′-deoxyuridine
DMED: Diabetes mellitus ED
NC: Negative control
Ad-Luc: Adenovirus-Luciferase
DMEM: Dulbecco’s modified Eagle’s medium
PBS: Phosphate buffer solution
DPBS: Dulbecco’s phosphate-buffered saline
IHC: Immunochemistry
WB: Western blot
SD: Sprague–Dawley
STZ: Streptozotocin
FBS: Fetal bovine serum
ICP: Intracavernosal pressure
MAP: Mean arterial pressure
APO: Apomorphine
CCSM: Corpus cavernosum smooth muscle
CCSMCs: Corpus cavernosum smooth muscle cells
MSCs: Mesenchymal stem cells
PDE5i: Phosphodiesterase type 5 inhibitor
PLGA: Poly l-glutamic acid
SML: Smooth muscle-like
