# Supplementary material for: In vivo tracking on longer retention of transplanted myocardin gene-modified adipose-derived stem cells to improve erectile dysfunction in diabetic rats
Source: Stem Cell Res Ther. 2019 Jul 16;10:208. doi: 10.1186/s13287-019-1325-7 (PMC6636019; doi:10.1186/s13287-019-1325-7)
Supplement: Supplementary file 1 — Table S1. Primer sequences used in this study. The forward and reverse primer sequences of β-actin, PCNA, α-SMA, Calponin, myocardin, SRF, SOX2, and OCT4 are shown in Table S1. (DOCX 16 kb) [file 13287_2019_1325_MOESM1_ESM.docx]

**In vivo tracking on longer retention of transplanted** **myocardin gene-modified adipose-derived stem cells to improve erectile dysfunction in diabetic rats.**

Hai-Bo Zhang^1#^, Feng-Zhi Chen^2#^, Shu-Hua He^1#^, Yan-Bing Liang^3^, Zhi-Qiang Wang^4^, Li Wang^1^, Ze-Rong Chen^1^, Wei Ding^5^, Shan-Chao Zhao^1*^, An-Yang Wei^1*^.

**Table S1**

**Primer sequences used in this study**

| Primer | Sequences |
| --- | --- |
| β-actin | F: GATCAAGATCATTGCTCCTCCTG  R: AGGGTGTAAAACGCAGCTCA |
| PCNA | F: AGTTTTCTGCGAGTGGGGAG  R: TCTACAACAAGGGGTACATCTGC |
| α-SMA | F: TTCAATGTCCCTGCCATGTA  R: CATCTCCAGAGTCCAGCACA |
| Calponin | F: ATCATTGGCCTACAGATGGGC  R: AGCGTGTCACAGTGTTCCAT |
| Myocardin | F: CTTGCAGATGACCTCAACGA  R: TCACGGAAGAATCCATAGGC |
| SRF | F: ACCAGCTTCACTCTCATGCC  R: TGCATGGGGACTAGGGTACA |
| SOX2 | F: CAAAAACCGTGATGCCGACT  R: TTCATCGCCCGGAGTCTAGT |
| OCT4 | F: GAGAGGGATGTGGTTCGAG  R: CCTCAGGAAAAGGGACCGAG |
