# Supplementary figures and images for: In vivo tracking on longer retention of transplanted myocardin gene-modified adipose-derived stem cells to improve erectile dysfunction in diabetic rats
Source: Stem Cell Res Ther. 2019 Jul 16;10:208. doi: 10.1186/s13287-019-1325-7 (PMC6636019; doi:10.1186/s13287-019-1325-7)

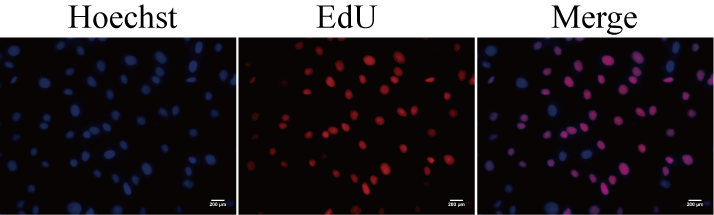

Supplement: Supplementary file 2 — Figure S1. EdU transfection efficiency was confirmed. Passage 2 ASCs were incubated with 10 mM EdU for 24 h before intracavernous injections and 81.53 ± 1.42% of cells were stained red under × 400 magnification. Scale bar = 200 μm. (TIF 608 kb) [file 13287_2019_1325_MOESM2_ESM.tif]
